# The Central Funds and the Special Hospitals

**Published:** 1905-01-14

**Authors:** 


### THE CENTRAL FUNDS AND THE SPECIAL HOSPITALS.

In its issue of the 11th instant the *Medical Press* has another article on this subject which will not add to its authority, we fear, as an exponent of accurate facts and opinions.

This article contains two misstatements—(1) that the Sunday Fund Council consists of fifty clerical or lay members, when in fact it numbers one hundred, of whom fifty are clerical and fifty lay members. (2) That the Fund has no representatives from the small hospitals, either upon its General Council or upon the Distribution Committee. As a matter of fact, both the honorary secretaries, who are members of the Council and of the Distribution Committee, are representatives of special hospitals, Mr. Martin, of the St. Mark's Hospital for Fistula, and Mr. Gilliat, of the Royal Chest Hospital. But the *Medical Press* may argue that virtue in a representative of small hospitals entails election by an Orthopædic Hospital, in view of the writer's evident interest in this speciality. Although, therefore, we have not space to set forth all the representatives of special hospitals on the Sunday Fund Council, we may also mention Mr. J. R. Cooper, who is the Chairman, we believe, of the Committee of the amalgamated Royal and National Orthopædic Hospitals. The truth seems to be, as this last article in the *Medical Press* shows on the face of it, that the writer has not made himself sufficiently familiar with the facts, or much of it could never have passed the editorial eye. Otherwise it would not have been contended that the constitution of the Hospital Sunday Fund is defective, on the strength of statements (1) and (2), which we have shown to have no foundation in fact.

The writer is equally at fault when he proceeds to invite discussion on other matters, for his so-called facts are again without foundation. He exhibits, too, a fatal disability to avoid restating, as facts, statements which we have proved to be without foundation, as for instance those relating to the sale of the site of the Royal Orthopædic Hospital. The documentary evidence published in these columns on this question proved the writer to be wrong, and finally disposed of the point raised.

Sites, however, appear to have a fascination for the writer in the *Medical Press* who this week proceeds to criticise the King's Fund, and in justification states :—

"The King's Fund pressed the sale of their former site on the Royal Ophthalmic (Moorfields) Hospital, a step that was followed by financial ruin." Now the King's Fund was not started until 1897, and the Royal London Ophthalmic Hospital purchased the site in the City Road, which involved the sale of the Moorfields site, on September 22nd, 1892, *or five years before the King's Fund was heard of.* What is to be said of a writer who perpetrates an absurdity of this kind, and then appeals to the members of the Royal Family to admire his courage for basing his criticisms upon it? At any rate our contemporary must admit that its wish has been gratified on the present occasion by the success which has attended its efforts "to obtain adequate published answers to its contentions."

